# Biofilm Formation and Detachment in Gram-Negative Pathogens Is Modulated by Select Bile Acids

**DOI:** 10.1371/journal.pone.0149603

**Published:** 2016-03-18

**Authors:** Laura M. Sanchez, Andrew T. Cheng, Christopher J. A. Warner, Loni Townsley, Kelly C. Peach, Gabriel Navarro, Nicholas J. Shikuma, Walter M. Bray, Romina M. Riener, Fitnat H. Yildiz, Roger G. Linington

**Affiliations:** 1 Department of Chemistry and Biochemistry, University of California Santa Cruz, Santa Cruz, CA 95064, United States of America; 2 Department of Microbiology and Environmental Toxicology, University of California Santa Cruz, Santa Cruz, CA 95064, United States of America; 3 Chemical Screening Center, University of California Santa Cruz, Santa Cruz, CA 95064, United States of America; 4 Department of Chemistry, Simon Fraser University, Burnaby, BC V5A 1S6, Canada; Institut Pasteur, FRANCE

## Abstract

Biofilms are a ubiquitous feature of microbial community structure in both natural and host environments; they enhance transmission and infectivity of pathogens and provide protection from human defense mechanisms and antibiotics. However, few natural products are known that impact biofilm formation or persistence for either environmental or pathogenic bacteria. Using the combination of a novel natural products library from the fish microbiome and an image-based screen for biofilm inhibition, we describe the identification of taurine-conjugated bile acids as inhibitors of biofilm formation against both *Vibrio cholerae* and *Pseudomonas aeruginosa*. Taurocholic acid (**1**) was isolated from the fermentation broth of the fish microbiome-derived strain of *Rhodococcus erythropolis* and identified using standard NMR and MS methods. Screening of the twelve predominant human steroidal bile acid components revealed that a subset of these compounds can inhibit biofilm formation, induce detachment of preformed biofilms under static conditions, and that these compounds display distinct structure-activity relationships against *V*. *cholerae* and *P*. *aeruginosa*. Our findings highlight the significance of distinct bile acid components in the regulation of biofilm formation and dispersion in two different clinically relevant bacterial pathogens, and suggest that the bile acids, which are endogenous mammalian metabolites used to solubilize dietary fats, may also play a role in maintaining host health against bacterial infection.

## Introduction

Biofilms are surface-attached bacterial communities encased in a matrix of exopolysaccharides, proteins, and extracellular DNA.[1, 2] It is estimated that biofilm formation contributes to persistence and virulence of up to 80% of microbial infections in the human body and of many hospital-acquired infections, particularly in cases when in-dwelling medical devices are required.[3–5] Biofilm-associated bacteria have been demonstrated to possess 10–1,000 fold greater resistance to antibiotic treatment compared to planktonic cells, making established biofilm infections particularly difficult to eliminate.[5] Biofilms are also important for environmental survival, transmission and increased infectivity of human pathogens.[6] Despite this, there are currently no clinically approved small molecule biofilm inhibitors on the market in the United States.

It has long been recognized that the disruption of biofilm formation and persistence is an attractive target for therapeutic intervention.[7] This target area has received significant attention in recent years, leading to the discovery of biofilm inhibitors for many of the commonly encountered bacterial pathogens.[8, 9] However, few of these compounds show activity against *V*. *cholerae*, prompting us to explore niche environments for new inhibitors of *V*. *cholerae* biofilm formation.

Using our image-based screen for biofilm inhibition [10, 11] we screened a library of bacterial extracts from the fish microbiome [12] and identified taurocholic acid (TCA, **1**) as a selective biofilm inhibitor in *Vibrio cholerae*. Taurocholic acid is a component of bile, which is composed of lipid molecules (cholesterol, phospholipids) and bile acids (a complex mixture including **1**, **5**, **9–11**). Bile acid concentration is estimated to be 0.2 to 2% in the intestine, [13] and is known to be important for the solubilization and transport of dietary fats.[14] In addition, bile acids are involved in the regulation of lipid, glucose, and energy metabolism, and are known to mediate drug metabolism and detoxification, [15] indicating a central role for bile acids in maintaining gut health.

In this study, screening of 12 major endogenous human bile acids as individual constituents revealed that a select subset of these compounds are capable of both inhibition and dispersal of biofilms in *V*. *cholerae* and *Pseudomonas aeruginosa*, and demonstrated a clear but different structure activity relationship (SAR) against each pathogen. Evaluation of antibiotic activities indicated that these compounds were selective disruptors of biofilm formation, without effect on bacterial cell survival. Additionally, we have screened the other components of bile, including various fatty acids, bilirubin, phosphocholine and cholesterol and determined that the observed activities are unique to the bile acids. These results suggest that bile acids may play a role in controlling infections by biofilm-forming pathogens, and demonstrate that this phenomenon is selective for specific bile acid/ pathogen combinations.

## Results and Discussion

### Discovery of Biofilm Inhibitors from a Fish Microbiome Natural Products Library

Recently, microbial symbionts from higher order organisms have been shown to have the capacity to produce a number of bioactive small molecules.[16] Specifically, biosynthetic gene cluster analyses have revealed a large potential for the production of natural products from gut associated microbes from the human gut microbiome.[17] Due to these observations, we previously investigated the natural products producing capacity of bacterial isolates from the fish microbiome for the presence of compounds with antibiotic activities against a panel of human and fish bacterial pathogens.[12] We noticed that a number of extracts possessed antibacterial activity towards both human and ichthyopathogenic *Vibrio* species, several of which are known to target fresh water and marine fish species.[18] In this study we now demonstrate that an extract of a bacterial culture from the fish microbiome also possess anti-biofilm activity against the Gram-negative human pathogens, *V*. *cholerae* and *P*. *aeruginosa*.

Prefractionated extracts from the fish microbiome library [12] were screened for biofilm inhibition using a 384-well plate high content screen which employs a rugose variant strain of *V*. *cholerae* that is chromosomally tagged to constitutively express GFP.[11] Screening of 147 prefractions identified one active fraction (FI1021D) that potently inhibited biofilm formation. Large-scale liquid culture of the fish microbiome-derived bacterium *Rhodococcus erythropolis* sp. FI1021, followed by extraction, bioassay-guided fractionation on a C_18_ SPE cartridge and final purification by a combination of HPLC and silica gel column chromatography afforded one bioactive pure compound. The activity was confirmed by final dilution series biological screening, and the identity of this bioactive metabolite determined as taurocholic acid (TCA, **1**) through a combination of 1D and 2D NMR and MS experiments.[19] This assignment was subsequently verified by comparison of the natural product to a commercial standard of **1**, which showed excellent agreement for ^1^H and ^13^C NMR shifts, HPLC retention time through co-injection, and optical rotation values (isolated taurocholic acid: [α]_D_^20^ +38.0; commercial taurocholic acid: [α]_D_^20^ +38.4), (S1 and S2 Figs).

The isolation of **1** from *R*. *erythropolis* sp. FI1021 represents only the second ever *de novo* isolation of this compound from a microbial source. The only other TCA-producing organism is also a marine-derived actinobacterium (*Aeromicrobium halocynthiae*) which, interestingly, was also isolated as an associant of a higher order organism (the ascidian *Halocynthiae roretzi*).[20] Specific bile acids have been previously isolated from fermentation broths of environmentally-derived bacteria, but with the exception of *R*. *erythropolis* sp. FI1021 and *A*. *halocynthiae*, none of these isolations produced taurine-conjugated bile acids, instead producing only cholic acid (**9**), deoxycholic acid (**11**), glycocholic acid (**5**), and glycodeoxycholic acid (**7**).[20–23]

To date, fish are mainly credited with the capability to synthesize C_27_ bile alcohols and C_24_ bile acids, with **9**, **10**, and 5α-cyprinol-27-sulfate being the most abundant endogenous bile components isolated from different fish species.[24] By contrast, **1** is an endogenous human metabolite that is a primary component of bile.[14] Typically bile acids are synthesized by the liver, stored in the gallbladder, and secreted into the small intestine after meals to assist in the elimination of cholesterol and the solubilization and transport of dietary fats. It is therefore possible that the relationship between *R*. *erythropolis* sp. FI1021 and its host is synergistic, with *R*. *erythropolis* sp. FI1021 producing non-endogenous bile acids to mediate biofilm-related infection in the fish gut, although this has not been proved experimentally.

### Expansion of the Evaluation of Biofilm Inhibition in Gram Negative Pathogens by Individual Bile Constituents

**Fig 1:**
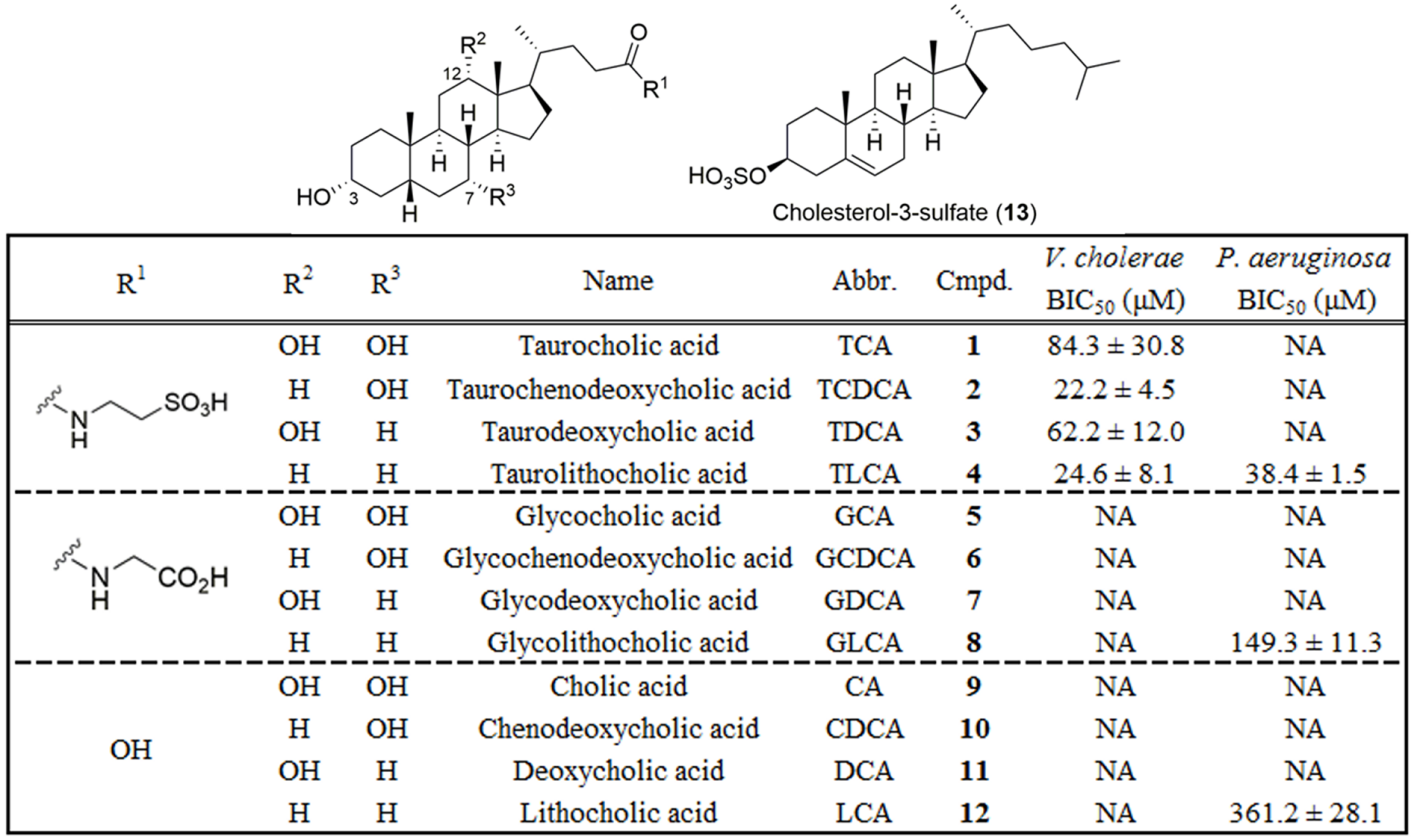
Structures and screening results for individual bile acids in both *V*. *cholerae* and *P*. *aeruginosa* biofilm inhibition assays. BIC_50_ = Biofilm Inhibitory Concentration required to reduce biofilm coverage by 50%. NA = not active.

Following the discovery of **1** as a biofilm inhibitor for *V*. *cholerae* we examined 12 endogenous human bile acid derivatives (**1–12**), as well as other components of bile including intact ox bile, fatty acids, bilirubin, and phosphocholine, and cholesterol for biofilm inhibition in both the *V*. *cholerae* and *P*. *aeruginosa* assay systems (Fig 1, S4 Table). Both pathogens are exposed to bile during infection, and it is known that *V*. *cholerae* utilizes intestinal bile as an *in vivo* signal to induce virulence factor production.[25] By contrast, *P*. *aeruginosa* encounters bile acids via aspiration of gastro esophageal reflux.[26] In addition to TCA (**1**), there are 11 other bile acids that are commonly encountered in the human digestive system (Fig 1). These 12 bile acids vary in both oxygenation pattern and side chain constitution (Fig 1). For each side chain (carboxylic acid, glycine or taurine) there are four oxygenation patterns, each of which possess a β-hydroxyl group at the 3-position, and vary by the presence or absence of β-hydroxyl groups at the 7 and 12-positions of the B and C rings of the steroid core. This high degree of structural similarity prompted us to examine the other 11 human bile acids for biofilm inhibition, using cholesterol 3-sulfate (C3S, **13**) as a non-bile acid control.

Dilution series of each bile acid (1 mM– 6 μM) were screened in triplicate in our standard image-based biofilm assay under static culture conditions, incubating for 4.5 h (*V*. *cholerae*) or 8 h (*P*. *aeruginosa*). Results from this screen revealed that for *V*. *cholerae* only those compounds containing the taurine side chain were active as biofilm inhibitors, with the oxygenation pattern on the steroid core having only a modest effect on potency. Of the four taurine conjugated bile acids screened against *V*. *cholerae*, TCDCA (**2**) and TLCA (**4**) were the most potent analogues, with biofilm inhibitory concentrations (BIC_50_) of 22.2 and 24.6 μM respectively (Fig 1 and S3 Fig). Surprisingly, when screened against *P*. *aeruginosa*, an entirely different SAR pattern was observed, driven by the oxygenation pattern on the steroid core, with only modest effects from substitutions of the side chain. In the *P*. *aeruginosa* assay the lithocholic derivatives TLCA (**4**) and GLCA (**8**) possessed the most potent biofilm inhibitory activities, with BIC_50_ values of 38.4 and 149.3 μM respectively (Fig 1 and S5 Fig). These discrete SAR patterns against different Gram-negative pathogens raises the possibility that the bile acids function independently to modulate biofilm formation for a range of microbial pathogens, with selected members of the bile acid family having activity against specific biofilm-forming microbes.

The role of bile and bile salts on biofilm formation is a complex and rapidly developing area of research. As of January 2016, a Pubmed search for the terms ‘bile’ and ‘biofilm’ returned 43 hits related to the impact of bile on biofilm formation. These manuscripts examined the impact of bile on biofilm development for a range of pathogenic and environmental bacterial strains, including *P*. *aeruginosa*, [26] *Klebsiella pneumoniae*, [27, 28] *Escherichia coli*, [29–31] *V*. *cholerae*[32–38] and other *Vibrio* species.[39–43]

The original study in this area was published by Costerton and co-workers in 1994. This work examined how the presence of two different bile acids (TCA and TDCA) effected the ability of three patient-derived Gram-negative pathogens (two strains of *E*. *coli* and one strain of *Enterococcus fecalis*) to adhere to polyethylene discs.[31] The objective of this study was to determine whether bile components altered the ability of these pathogenic strains to adhere to the plastic material used in the fabrication of biliary stents. The authors concluded that the effect of bile salts on biofilm was strain-dependent, and that TDCA but not TCA was capable of reducing biofilm formation for the two *E*. *coli* strains when included in the culture medium at 25 or 50 mM.

Following this initial report, a number of other publications have explored the role of either intact bile or individual bile acid constituents on biofilm development and progression. In some of these cases, bile has been reported as an antimicrobial, [32, 44] whereas in other cases bile is reported to have no impact on microbial growth.[26, 41, 43, 45] In a similarly confusing situation, many of these papers provide evidence that the presence of either intact bile or individual bile acids increase biofilm formation [26, 32, 34–36, 40–42, 45–52] whereas a number of other papers report that addition of bile or individual bile acids *decreases* biofilm formation.[26, 30, 31, 33, 51, 53]

This seemingly incompatible set of results can perhaps best be explained by more careful examination of the experimental conditions employed in each case. In situations where researchers examined multiple genera, it was noted that the impact of bile on biofilm formation was highly species-dependent, and that even strains of the same species displayed markedly different biofilm forming abilities.[26, 31, 42] Furthermore, it is clear that both the composition of bile salts used, and the concentrations at which they are employed are both key factors that impact biofilm formation. Often, bile or bile acids were applied at a single concentration, making it difficult to directly compare these data. In one instance, it has been reported that bile can increase biofilm formation at low concentrations, while inhibiting biofilm formation at higher concentrations, further complicating this issue.[51] Added to this, it has been shown that varying media components also alters the effect of bile on biofilm formation, [49] providing another variable that must be considered when comparing the results of different studies in this area. Finally, it is widely acknowledged that biofilm formation includes a strong temporal progression, and that the application of bile acids for different amounts of time will also impact their effect on this process.[46]

Because intact bile can be obtained from a variety of commercial sources, it is quite likely that the individual bile acid constitution of these biological mixtures is not well conserved between lots. Given that we and others have demonstrated a very strong structure activity relationship (SAR) between bile acid structure and biofilm formation, it is plausible to suggest that some of the reported variation in the effects of intact bile can be attributed to variations in bile acid composition within these samples.

With respect to the current study, five previous papers have been published that directly examine the effects of intact bile or individual bile acids on biofilm formation in *V*. *cholerae*.[32–36] Of these, four report that intact bile causes an increase in biofilm biomass when treated for 1–24 hours.[32, 34–36] Replication of these experiments in our own laboratory using the A1552 and C6706 strains of *V*. *cholerae* and commercial ox bile indicated that for both of these strains, intact bile reduced rather than increased biofilm coverage when treated for a range of timepoints (S4 Fig). By contrast, the only previous study to examine the effects of individual bile acids (CA, DCA, CC and TCA; 1 mM) on *V*. *cholerae* biofilm formation revealed strong inhibitory activity selectively for the taurine-conjugated bile acid TCA. This is in line with our results, which reveal that for the 12 most prevalent bile acids found in the human gut only those with the taurine-conjugated side chain are capable of inhibiting biofilm formation in *V*. *cholerae* O1, El Tor A1552.

Given that prior reports have presented the impact of intact bile on biofilm development, [33, 34] other components of bile were also assayed for biofilm inhibition including bilirubin, fatty acids, and phosphocholine. None of these components were found to inhibit biofilm formation up to the highest soluble concentration in DMSO in our 384-well plate screen (S4 Table). For subsequent experiments the most potent analogue from primary screening was selected (Fig 1). For *V*. *cholerae* we therefore studied compound **2**, and compared its activity to the original natural product TCA (**1**). For *P*. *aeruginosa* we studied compound **4**, but excluded the original natural product because TCA was demonstrated to have no activity in the original *P*. *aeruginosa* screen (Fig 1).

### Validation of the Fish Microbiome-Derived Natural Product 1, and Compound 2 as Biofilm Inhibitors against *V*. *cholerae*

**Fig 2:**
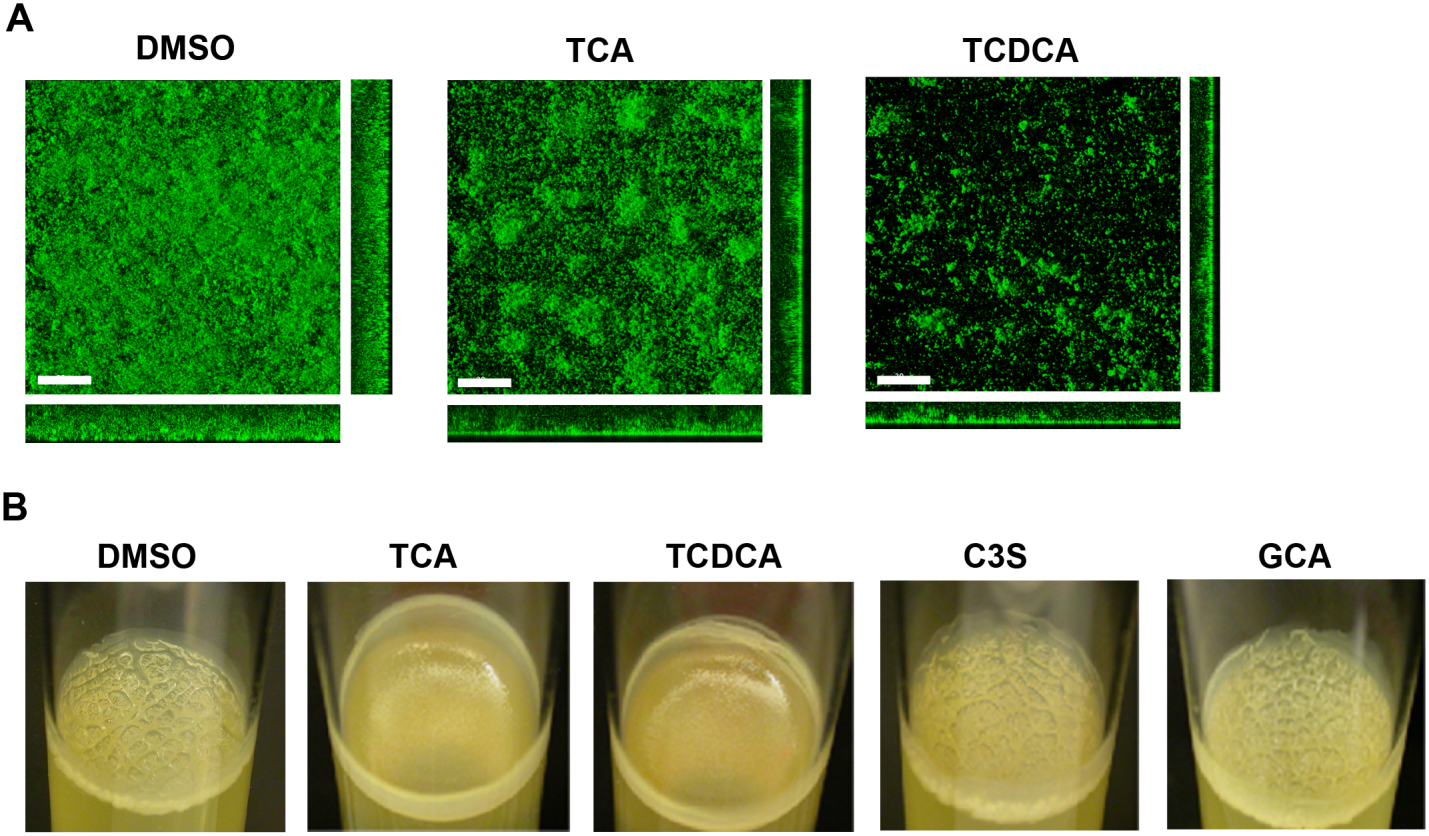
Bile acid components reduce biofilm formation in *V*. *cholerae*. (A) CLSM images of horizontal (xy) and vertical (xz and yz) projections of biofilm structures formed by the wild-type strain (wt) in the presence of DMSO as a control and 200 μM of TCA or TCDCA. Biofilms were incubated at 30°C and images were taken at 24 hours. (B) Pellicle formed by the rugose strain formed after 2 days of incubation at 30°C with different bile acid components. All assays were repeated with two biological replicates.

To validate the primary screening results, the effects of compounds **1** and **2**, as well as control compounds **5** and **13**, were assessed for their effects on biofilm formation in *V*. *cholerae* under static conditions. Biofilms formed in the presence of test compounds or vehicle controls were imaged 24 h post-inoculation using confocal scanning laser microscopy (CSLM) and quantified using COMSTAT analysis. Both **1** and **2** displayed significant inhibition of biofilm formation, with reductions in total biomass at 24 h of 3.7 and 5.7-fold for **1** and **2** respectively (Fig 2A and S1 Table) compared to vehicle-only control (DMSO). Similarly, control compounds **5** and **13** did not inhibit biofilm formation at tested concentrations.

We also analyzed the impact of bile acids on pellicle biofilms, formed at the air-liquid interface. The rugose strain forms corrugated pellicles due to its enhanced ability to produce exopolysaccharide *Vibrio* polysaccharide (VPS) and biofilm matrix proteins. When pellicles were formed in the presence of the bile acids **1** and **2**, they lacked corrugation, suggesting that biofilm matrix production was decreased. In contrast, **5**, **13** and vehicle only controls did not impact pellicle morphology (Fig 2B).

We determined that **1** does not impact cell growth up to the highest tested concentrations (1 mM) using both OD_600_ measurements and plating assays, which indicates that reduction in biofilm coverage is not due to decreased growth or reduction in cell-viability (S3 Fig). In physiological settings, bile acids are known to form micelles to transport dietary fats.[14] However, in the static assay system, only the bile acids which had shown activity in the initial assay (**1**, **2**) recapitulated the biofilm inhibition phenotype, while the other bile acid (GCA, **5**) and cholesterol-3-sulfate (C3S, **13**) where completely inactive, indicating that the observed biofilm inhibition is structure dependent, and therefore not the result of detergent effects.

It has previously been reported that treatment with intact bile induced biofilm formation and biofilm gene expression in the *V*. *cholerae* strain C6706.[34] However, transcriptome analysis of *V*. *cholerae* cells exposed to intact bile was separately reported not to significantly alter the expression levels of genes involved in biofilm formation (*vps* genes and VpsR), albeit under conditions not explicitly designed to induce biofilm formation.[54] Furthermore, a recent report indicates that taurocholic acid is capable of dispersing mature biofilms at physiologically relevant concentrations.[33] Therefore it is considered that the role of bile acids in the mediation of biofilm formation and persistence remains a contested issue.[55] In our own laboratory, examination of intact bile and individual bile constituents using the crystal violet biofilm quantification method employed in previous studies did not lead to induction of biofilm formation, and in several instances significantly reduced biofilm formation, in line with our image-based screening results (S4 Fig).

**Fig 3:**
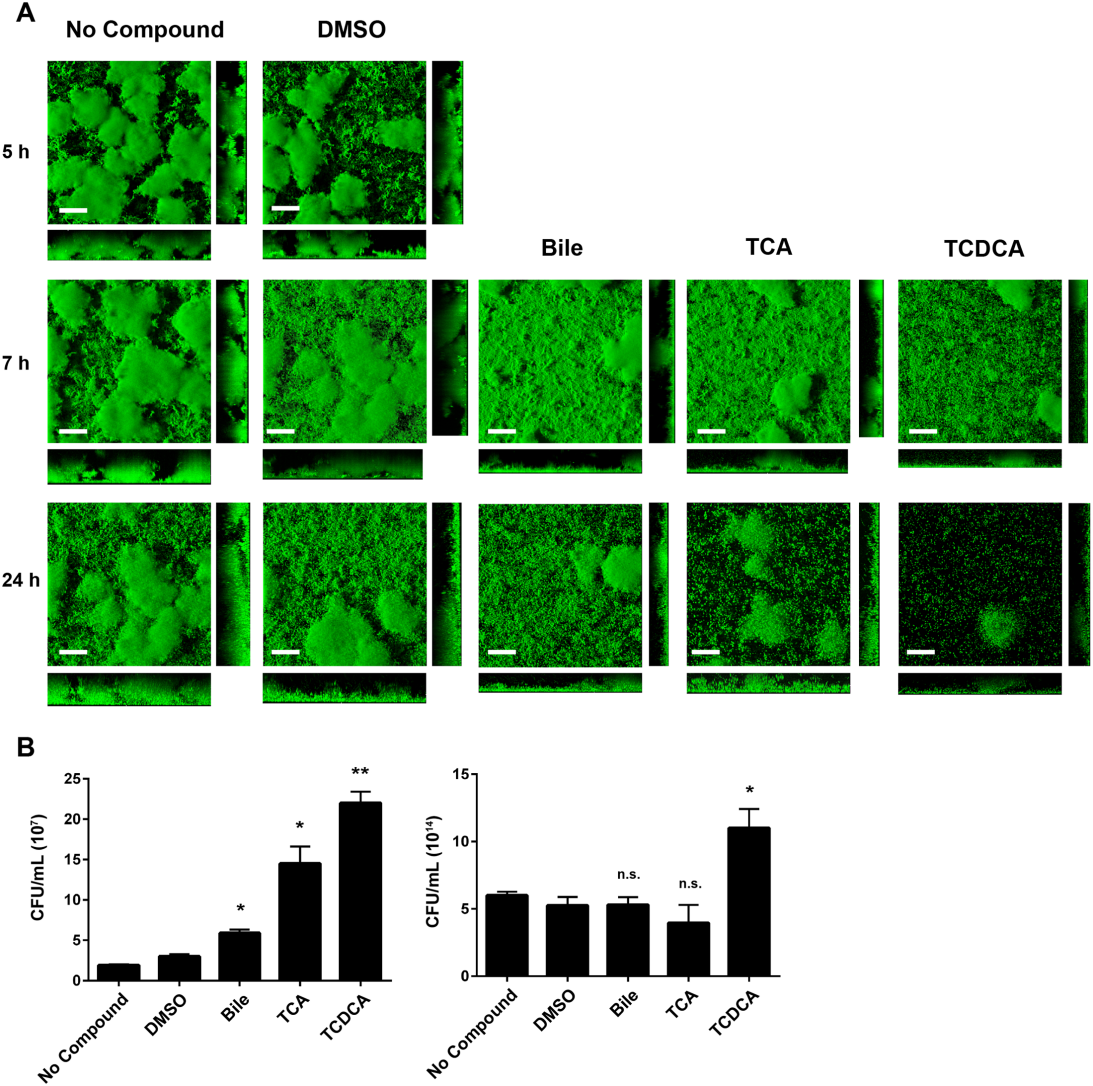
Bile acid components induce detachment of *V*. *cholerae* biofilms. (A) CSLM images of horizontal (xy) and vertical (xz and yz) projections of biofilm structures formed by the wild-type strain (wt). Biofilms were formed at 30°C for 5 hours. These biofilms were either untreated or exposed to DMSO, 0.4% Ox bile, 150 μM of TCA or TCDCA. CSLM images of biofilms were taken at 5, 7, and 24 hours. (B) Detachment from biofilms was evaluated by quantification of the planktonic population. CFU/mL of cells released from biofilms to the planktonic population was quantified for each condition at 7 h (left) and 24 h (right). Error bars indicate standard deviations of three biological replicates. *P < 0.05, **P < 0.005, n.s., *P* > 0.05.

Humans ingest *V*. *cholerae* biofilms during infection. Previous studies have demonstrated that biofilms are dispersed during colonization of the small intestine by bile acids.[16, 17] To document the impact of bile acids to biofilm structure, we evaluated the effects of **1** and **2** using a preformed biofilm dispersal model (Fig 3). For these experiments biofilms were grown in the absence of compound or in the presence of the vehicle only control for 5 h, after which inhibitors (150 μM) were added to biofilm growth medium. Addition of intact bile was also included for comparison. Biofilms were imaged using CSLM at 5 h (pre-compound addition), 2 h, and 19 h post-compound addition, then analyzed using COMSTAT. Additionally, the number of planktonic cells from each condition was quantified by calculating CFU/mL in media above biofilms. At all time points there was a slight decrease in biomass and thickness between the no compound and vehicle only control (Fig 3 and S3 Table). At both post-compound addition time points, **1** and **2** significantly decreased biofilm coverage compared to media and vehicle only controls. When compared with the vehicle only control, **1** and **2** reduced total biomass 2-fold and 2.8-fold respectively at 7 h, and 4-fold and 22-fold respectively at 24 h (Fig 3 and S3 Table). Additionally, planktonic CFU/mL was significantly increased in the presence of **1** at 7 h (*P* < 0.05) and **2** at both 7 h and 24 h (*P* < 0.05) (Fig 3B).

Dispersal of mature biofilms is in some ways a more important consideration than prevention of initial attachment for small molecule biofilm inhibitors. Treatment of nosocomial infections often requires the elimination of established biofilms, which can be difficult to achieve with existing antibiotics. A transcriptomic analysis of different ‘lifestyles’ exhibited by *P*. *aeruginosa* revealed dispersed cells represent a distinct lifestyle compared to planktonic or biofilm cells. These cells were more virulent than planktonic cells, but co-dosing with an iron chelator and an antibiotic was an effective form of treatment.[56] Antibiotic resistance is a commonly observed phenomenon for cells in the biofilm state.[5] Therefore the observation that **1** and **2** can impact the persistence of preformed biofilms is significant, in that it offers insight into one factor that may contribute to the complex regulatory mechanism of infection and transmission of cholera in the human host.

### Validation of 4 as a Biofilm Inhibitor against *P*. *aeruginosa*

**Fig 4:**
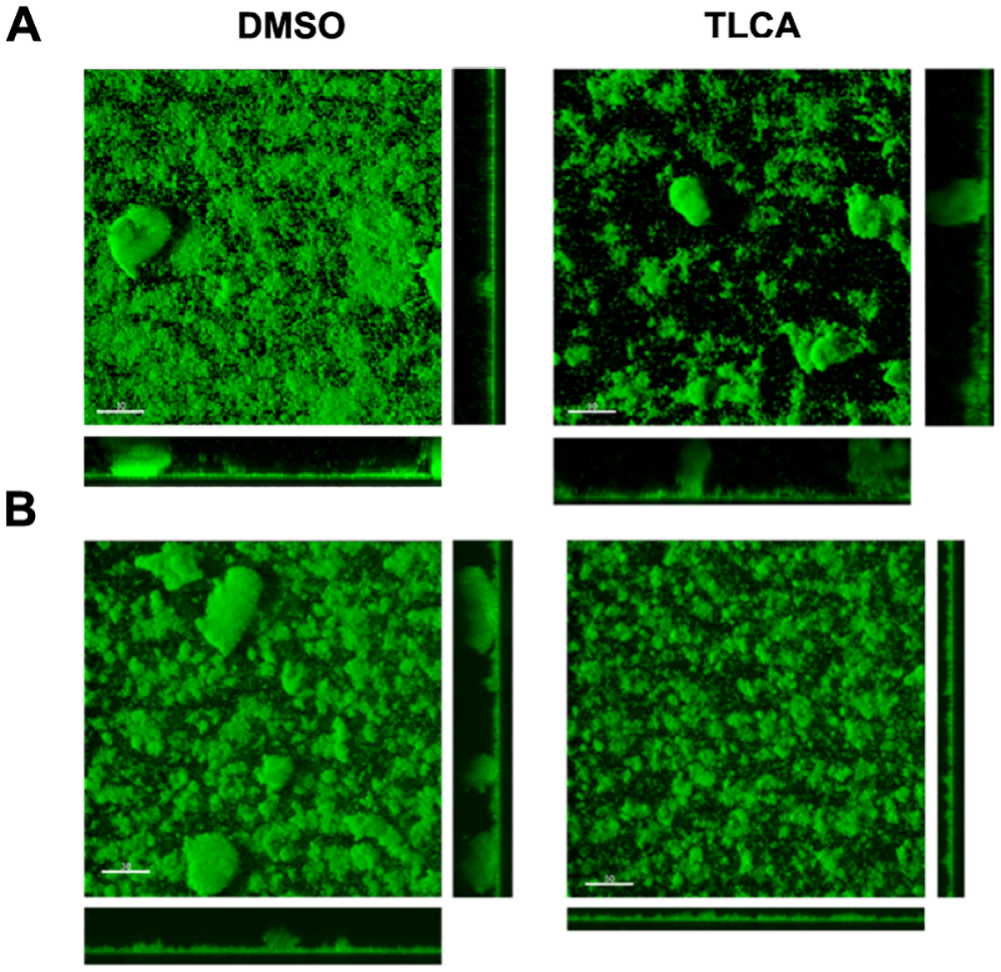
Bile acid components effect biofilm formation in *P*. *aeruginosa*. (A) CLSM images of horizontal (xy) and vertical (xz and yz) projections of biofilm structures formed by *P*. *aeruginosa* Δ*wspF* mutant in the presence of DMSO control or 50 μM of TLCA. Biofilms were incubated at 37°C and images were taken at 24 hours. (B) CSLM images of preformed biofilms after treatment with either DMSO control or 50 μM of TLCA for 19 hours at 37°C.

To analyze impact of TLCA (**4**) on *P*. *aeruginosa* biofilm formation, we examined biofilms formed after 24 h of incubation in the presence of 0.5% DMSO or 50 μM **4** using CSLM. *P*. *aeruginosa* biofilms grown in the presence of **4** had a 4.8-fold reduction in biomass at 8 h and a 1.7-fold reduction at 24 h compared to the vehicle control (Fig 4, S3 Table). In line with the observed effects in *V*. *cholerae*, addition of either **5** or **13** showed no impact on biofilm formation at either 8 or 24 h (data not shown). Cellular viability analysis measured through the addition of XTT demonstrated comparable bacterial growth with the DMSO control vehicle and demonstrate that, as with **1** and **2** in *V*. *cholerae*, **4** directly impacts biofilm formation and persistence in *P*. *aeruginosa*, rather than impacting cellular survival (S5 Fig).

### Possible Mechanisms of Action and Physiological Significance

Bile acids have been shown to possess a wide variety of biological activities.[15] They are critical for the digestive process and may protect a host against enteric pathogens.[13, 57] It is important to note that not only have enteric pathogens developed bile resistance mechanisms, but that they also utilize bile as a host signal to regulate virulence factor production.[25] It has previously been reported that *V*. *cholerae* exhibits significant changes to its transcriptome upon exposure to bile.[58] In particular, expression of resistance-nodulation-cell division (RND) family multidrug resistance (MDR) efflux pumps (*vexB*, *breB*) and transcriptional regulators *vexR* and *breR* were increased in response to bile.[59–61] *V*. *cholerae* also changes its outer membrane porin profile producing more OmpU and less OmpT which have small and large pore size, respectively and results in decreased bile uptake into the cell.[62, 63] Collectively these two changes in transport systems and efflux pumps mediate bile resistance in *V*. *cholerae*. A recent study reported that TCA does not impact biofilm gene expression, that the action of TCA does not require new protein synthesis, and the mode of action of TCA is likely to involve release of exopolysaccharide from biofilms.[33] However, the molecular details by which TCA leads to biofilm inhibition and dispersal are not fully understood.

Biofilm formation in both *V*. *cholerae* and *P*. *aeruginosa* depends on the production of biofilm matrix components exopolysaccharides, matrix proteins and nucleic acids and is highly regulated by transcriptional activators, repressors, alternative sigma factors, small RNAs, and responses to a variety of intracellular and extracellular signals. As such, the system is highly complex and influenced by an array of interrelated regulatory mechanisms, with the result that the mode of action of bile acids on biofilm inhibition remains unclear.

The discovery of selective bile acid constituents as modulators of biofilm formation in two different bacterial pathogens offers a number of implications in terms of physiological significance. Bile and bile acid constituents have been associated with a variety of physiological effects on gut/ microbe interactions, including the ability to modulate gut bacteria implicated in irritable bowel syndrome associated with high fat diets.[64] Given this, it is clear that bile acids play a much larger role in maintaining host health than simply solubilizing dietary fats. Concentrations of these compounds can reach concentrations of 1–14 mM when secreted from the gallbladder following meals, with most bile acids being recycled by the enterohepatic system rather than being newly synthesized by the body from cholesterol.[14] The concentrations tested in this study are well below physiological concentrations, suggesting that the observed activities could be important for intestinal health.

Taken together these results demonstrate that individual bile acids have distinct and selective effects on the ability of specific bacterial pathogens to form and maintain biofilm structures. Interestingly, these compounds are able to induce detachment of preformed biofilm colonies, placing them among a small number of compounds with similar activity profiles. These endogenous mammalian metabolites display biological activities in our *in vitro* biofilm models at well below the relevant physiological range, and are neither bactericidal nor cytotoxic, as confirmed by in house cytotoxicity screening (data not shown). We hypothesize that the mechanism by which the compounds work against these pathogens is likely conserved yet pathogen-specific, given that select derivatives demonstrated the same biological affect at similar concentrations in both Gram-negative pathogens but with distinctly different SAR patterns.

## Conclusion

In the era of the Human Microbiome Project, new roles for endogenous metabolites are constantly being uncovered. Multiple studies have implicated bile acids in the regulation of a variety of processes in physiological settings. Here we demonstrate that select bile acids are connected to modulation of biofilm formation, and therefore persistence, in both an enteric pathogen and a common nosocomial pathogen. The effective concentrations are well below the endogenous levels in the human host, suggesting that the bile acids may help prevent biofilms from forming in an environmentally relevant setting. These activities warrant further investigation in order to examine the structure activity relationship of the bile acid series against other enteric biofilm forming pathogens, as well as microbes that occur as part of healthy commensal gut microbial communities.

## Materials and Methods

### General Experimental Procedures

All commercially available reagents were used as received. Cholic acid, sodium taurodeoxycholate, sodium taurolithocholate, and glycochenodeoxycholic acid were purchased from Spectrum laboratory products. Sodium taurochenodeoxycholate, chenodeoxycholic acid, lithocholic acid, glycocholic acid, cholesterol 3-sulfate sodium salt, phosphocholine chloride sodium salt, and bilirubin were purchased from Santa Cruz Biotechnology. Deoxycholic acid, palmitic acid, stearic acid, arachidonic acid, linoleic acid, and oleic acid were purchased from VWR. Sodium taurocholate and cis-9-hexadecenoic acid were purchased from Fisher Scientific, sodium glycodeoxycholate was purchased from EMD, and dehydrated, purified ox bile was purchased from Sigma-Aldrich.

LC-MS analyses were conducted using either an Agilent 1200 series/6130 ESI single quadrupole LCMS, equipped with a Phenomenex Luna C_18_ (4.6 x 150 mm, 5μm) RP-HPLC column or an Agilent 1260 uPLC/6230 ESI-TOF equipped with a Jetstream source. All solvents were HPLC grade and were used without further purification. Optical rotations were measured on a Jasco P-2000 polarimeter using a 10 mm path length cell at 589 nm. NMR spectra were acquired on a Varian Inova 600 MHz spectrometer equipped with a 5 mm HCN triple resonance cryoprobe, and referenced to residual solvent proton and carbon signals (C_5_D_5_N, δ_H_ = 7.22, 7.58 and 8.74; δ_C_ = 123.8, 135.9, 150.4). High-resolution mass spectra were acquired with an ABI Mariner ESI-TOF-MS.

### Culture Conditions, Extraction, and Isolation

Seed culture of *R*. *erythropolis* sp. FI1021 was grown in 1 L of modified SYP broth [65] (1 L MilliQ water, 32.1 g Instant Ocean^™^, 10 g starch, 4 g peptone, 2 g yeast) with 20 g of Amberlite XAD-16 resin shaking at 200 rpm for 10 days at 27°C. Culture broth and resin slurries were filtered through glass microfiber filters, washed with water (3 x 200 mL) and the cells, resin, and filter paper extracted with 1:1 methanol/dichloromethane (250 mL). Organic fractions were dried *in vacuo* and subjected to solid phase extraction (SPE) using a Supelco-Discovery C_18_ cartridges (5 g) eluting with a step gradient of 40 mL of MeOH/H_2_O solvent mixtures (10% MeOH, 20% MeOH, 40% MeOH, 60% MeOH, 80% MeOH, 100% MeOH) and finally with EtOAc to afford seven fractions. The resulting fractions were dried *in vacuo*.

The active 60% MeOH fraction was subjected to C_18_ RP-HPLC Phenomenex Luna C18 (4.6 x 150 mm, 5 μm), 50–70% MeOH/ 50–30% H_2_O (acidified with 0.002% formic acid) over 50 min, 1 mL/min, 210 nm, t_R_ = 38.2. This RP-HPLC fraction was subjected to flash chromatography 5% MeOH/DCM acidified with 0.02% formic acid followed by a 100% MeOH wash acidified with 0.1% formic acid to afford TCA (**1**) as an optically active yellow solid.

Structure elucidation for isolated TCA (**1**) was performed as follows. ESI-TOF-MS analysis gave the molecular ion [M + Na]^+^ at 538.2812 (calcd for C_26_H_45_O_7_NSNa, 538.2814) and [2M + H]^+^ at 1031.5917 (calcd for C_52_H_91_N_2_O_14_S_2_, 1031.5911) and ESI-MS [M—H]^-^ at 514.2. In addition to these molecular ions, there were three consecutive losses of 18 Da that corresponded to the loss of three hydroxyl groups. The ^1^H NMR for this compound was also characteristic of a steroid type core with two large methyl singlets, complex signals in the methine and methylene regions, the presence of one carbonyl carbon, three oxygenated methines, and an unusual chemical shift at 50.0 in the ^13^C spectrum. Careful examination of these NMR data, coupled with interpretation of the mass spectral fragmentation led to the identification of the bioactive metabolite as TCA (**1**). NMR analysis, LCMS co-injections with a commercial sample and comparison of optical rotation values confirmed this assignment.

#### Isolated Taurocholic Acid (1)

^1^H NMR (C_5_D_5_N, 600 MHz) δ 4.21–4.20 (m, 2H), 4.09 (d, *J* = 2.4 Hz, 1H), 3.79–3.74 (m, 1H), 3.41 (t, *J* = 6.6 Hz, 2H), 3.36 (q, *J* = 7.2 Hz, 1H), 3.15 (q, *J* = 12.6 Hz, 1H), 2.93 (td, *J* = 4.8 Hz, 12.6 Hz, 12.6 Hz, 1H), 2.76–2.70 (m, 1H), 2.43–2.38 (m, 1H), 2.31–2.24 (m, 2H), 2.20–2.15 (m, 1H), 2.12–2.08 (m, 2H), 2.03–1.93 (m, 2H), 1.83–1.78 (m, 4H), 1.67–1.61 (m, 2H), 1.59–1.52 (m, 1H), 1.52–1.50 (m, 2H), 1.33–1.25 (m, 6H), 1.20–1.16 (m, 2H), 1.14 (d, *J* = 6.6 Hz, 3H), 1.07 (td, *J* = 3.6 Hz, 13.2 Hz, 13.2 Hz, 1H), 1.01 (s, 3H), 0.77 (s, 3H); ^13^C NMR (C_5_D_5_N, 150 MHz) δ13.4, 18.0, 23.6, 24.2, 27.7, 28.5, 30.0, 32.0, 32.7, 34.0, 35.7, 36.2, 36.4, 36.6, 36.7, 41.0, 41.4, 42.8, 43.0, 47.2, 47.4, 50.0, 68.0, 72.2, 72.7, 174.6; HRESITOFMS *m/z* [M + Na]^+^ at 538.2812 (calcd for C_26_H_45_O_7_NSNa, 538.2814).

#### Strains and Growth Conditions

*V*. *cholerae* O1, El Tor A1552, rugose variant (Fy_Vc_2) or *P*. *aeruginosa* PAO1 lacking *wspF* were used, as they have enhanced ability to form biofilms due to increased production of cyclic-di-GMP.[66, 67] Cultures were grown in LB media (1% tryptone, 0.5% yeast extract and 1% NaCl, pH 7.5) with aeration at 30°C for *V*. *cholerae* or 37°C for *P*. *aeruginosa* or on solid LB plates (1% agar).

#### Biofilm Inhibition Assay Protocol

Performed as previously described.[10, 11]

#### Biofilm and Pellicle Assays

Both *V*. *cholerae* and *P*. *aeruginosa* static growth chamber assays were performed using the same general protocol. For static biofilm chambers, overnight cultures were diluted 1:100 in fresh LB medium containing a final concentration of inhibitor (200 μM of either compound **1** or **2** for *V*. *cholerae*; 50 μM of compound **4** for *P*. *aeruginosa*), controls (GCA or cholesterol-3sulfate (C3S), 200 μM for *V*. *cholerae*, 50 μM for *P*. *aeruginosa*) or DMSO vehicle only. Biofilms were grown under non-shaking conditions at 30°C (*V*. *cholerae*) or 37°C (*P*. *aeruginosa*) in Lab-Tek II chambered coverglass slides (Nunc, NY).

For preformed static biofilm chambers, stable biofilm growth was demonstrated by diluting overnight cultures 1:100 in either fresh LB medium or LB with 1% DMSO and incubating these cultures in chambered coverglass slides for 5 h prior to imaging (Fig 3A). For assay chambers, overnight cultures were diluted 1:100 in fresh LB medium, incubated for 5h, and test compounds or DMSO vehicle controls added (150 μM in DMSO for *V*. *cholerae*, 50 μM in DMSO for *P*. *aeruginosa*; final DMSO concentration 1%). These test cultures were incubated for a further 2 h for the 7 h time point, or 19 h for the 24 h timepoint. Biofilms were washed twice with fresh LB medium and imaged. A serial dilution was performed using media above biofilms to plate and quantify planktonic cells. Confocal images of the biofilms that formed in static biofilm chambers were captured with a LSM 5 PASCAL laser scanning microscope at 488 nm excitation. Three-dimensional images of the biofilms were reconstructed using Imaris (Bitplane) and quantified using COMSTAT. The experiments were performed three times independently, and three images were taken for each biological replicate.

For analysis of pellicle formation, glass culture tubes (18 x 150 mm) containing 5 mL LB medium were inoculated with overnight-grown cultures, resulting in a 200-fold dilution. The tubes were incubated at 30°C under non-shaking conditions for 2 days. Assays were repeated with at least two different biological replicates. Statistical significance was determined using a two-tailed student’s t test.

#### Crystal Violet Biofilm Assay

Crystal violet biofilm assays were carried out as described previously.[68] Briefly, 96-well polyvinyl carbonate plates containing inhibitor, controls, or vehicles only were inoculated with 100 μL of 100-fold diluted overnight culture grown at 30°C. At selected time points after inoculation, unattached cells were removed and washed with distilled water. Attached biofilm was stained with 1% crystal violet at room temperature for 15 min. The amount of biofilm formed was quantified by dissolving the crystal violet in ethanol and comparing absorbance at 595 nm for test wells versus control wells.

## Supporting Information

S1 Fig NMR spectra for isolated and commercial samples of taurocholic acid (1), C_5_D_5_N, 600 MHz. (PDF)

S2 Fig ESI-TOFMS spectra for isolated and commercial samples of taurocholic acid (1). (PDF)

S3 Fig BIC_50_ curves and cellular viability as measured by OD_600_ for selected bile acids against *V*. *cholerae*. (PDF)

S4 Fig Crystal violet assay of biofilm formation in *V*. *cholerae* for intact bile and individual bile acid constituents. (PDF)

S5 Fig BIC_50_ curves and cellular viability as measured by OD_600_ for selected bile acids against *P*. *aeruginosa*. (PDF)

S1 Table COMSTAT analysis for bile acid components that reduce biofilm formation in *V*. *cholerae*. (PDF)

S2 Table COMSTAT analysis for *V*. *cholerae* preformed biofilm formation. (PDF)

S3 Table COMSTAT analysis for bile acid components that reduce biofilm formation in *P*. *aeruginosa*. (PDF)

S4 Table BIC_50_ screening results in *V*. *cholerae* for major non-bile acid components of bile. (PDF)
