# Supplementary material for: Biofilm Formation and Detachment in Gram-Negative Pathogens Is Modulated by Select Bile Acids
Source: PLoS One. 2016 Mar 18;11(3):e0149603. doi: 10.1371/journal.pone.0149603 (PMC4798295; doi:10.1371/journal.pone.0149603)

Figure S1. NMR Spectra for Isolated and Commercial Samples of Taurocholic Acid (**1**), pyridine-d5, 600 MHz.

^{1}H NMR spectra of TCA (1) isolated from extract *R. erythropolis* FI1021DH2S2

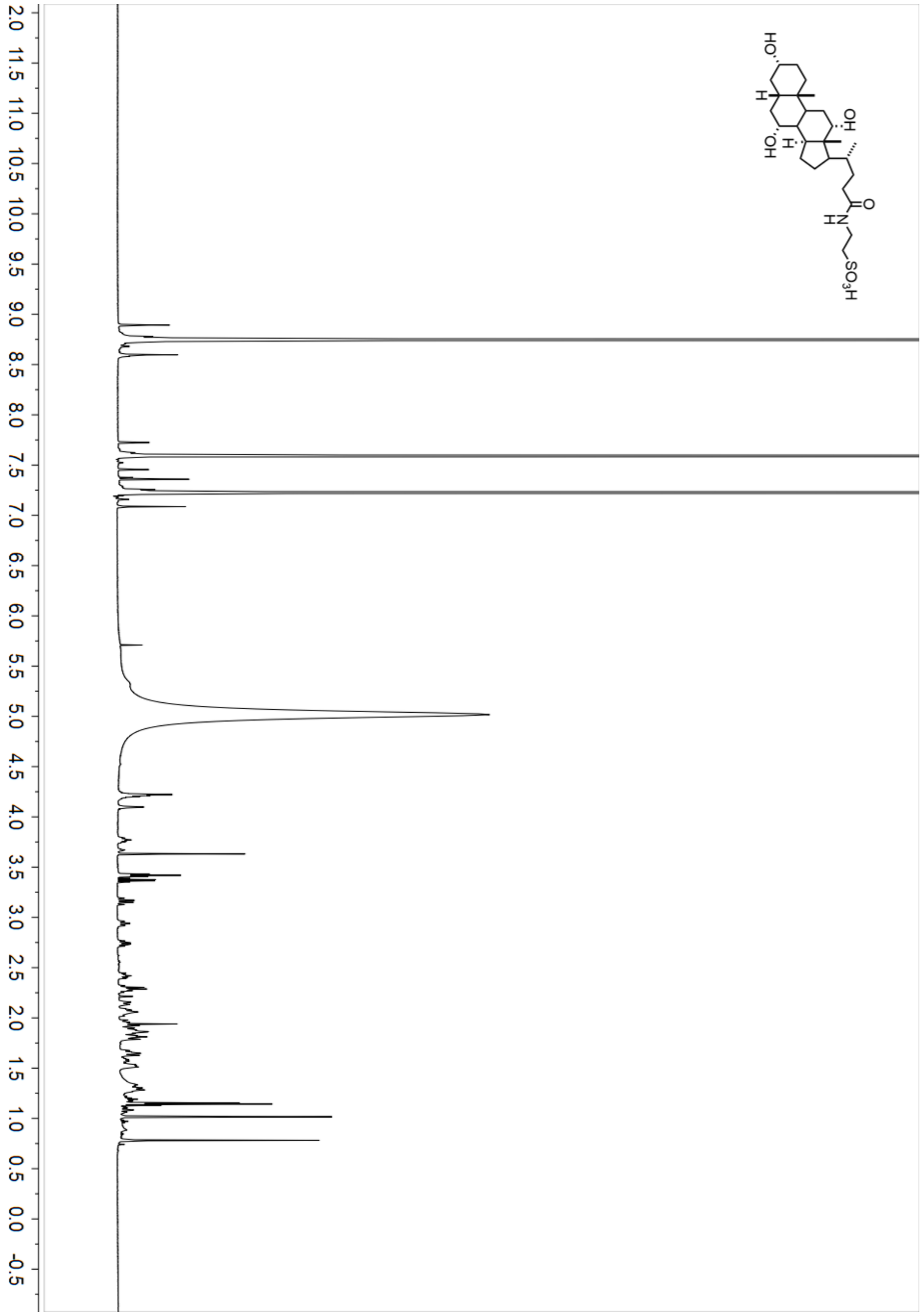

^{13}C NMR spectra of TCA (1) isolated from extract *R. erythropolis* FI1021DH2S2

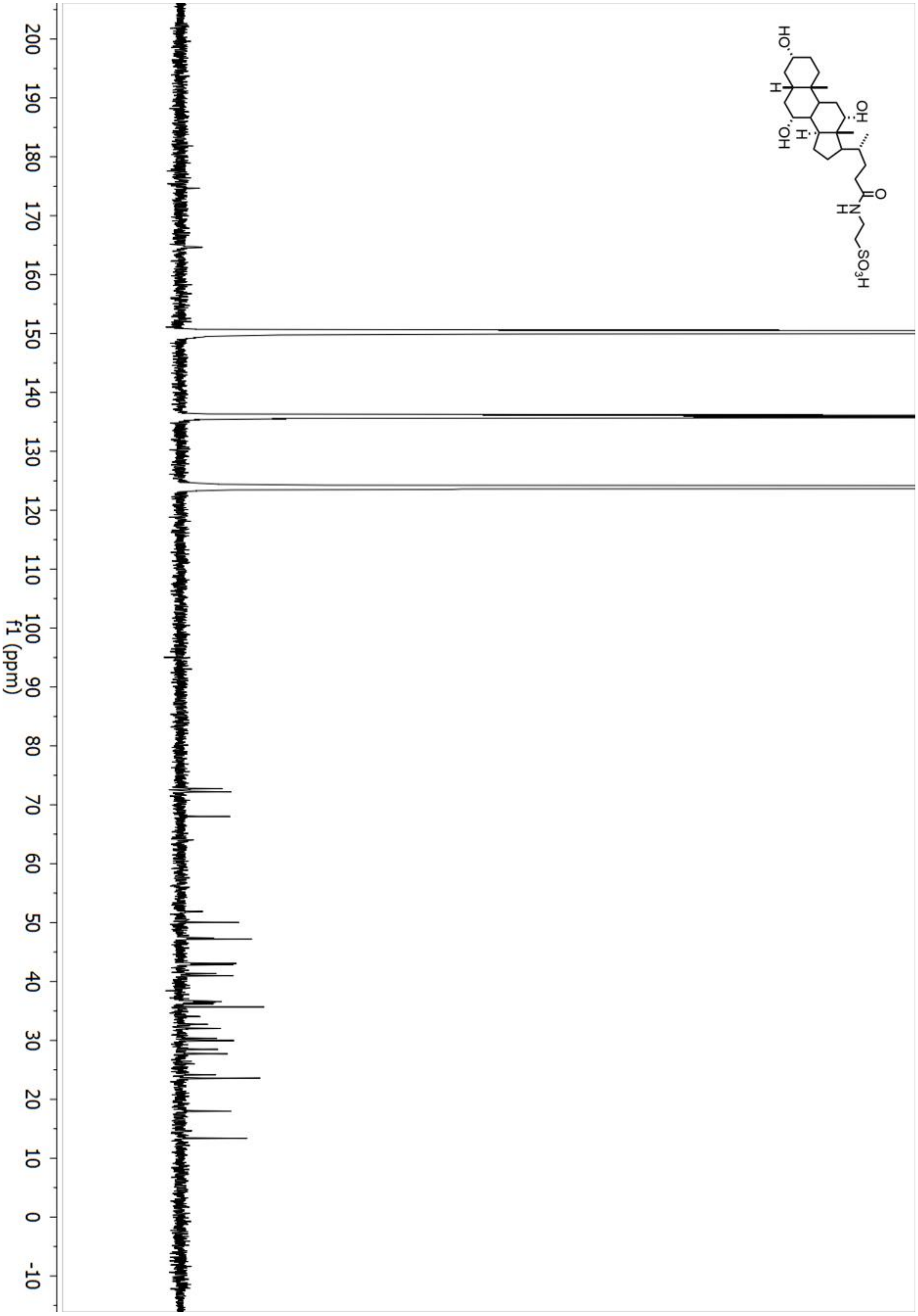

^{1}H NMR spectra of commercially purchased TCA (1)

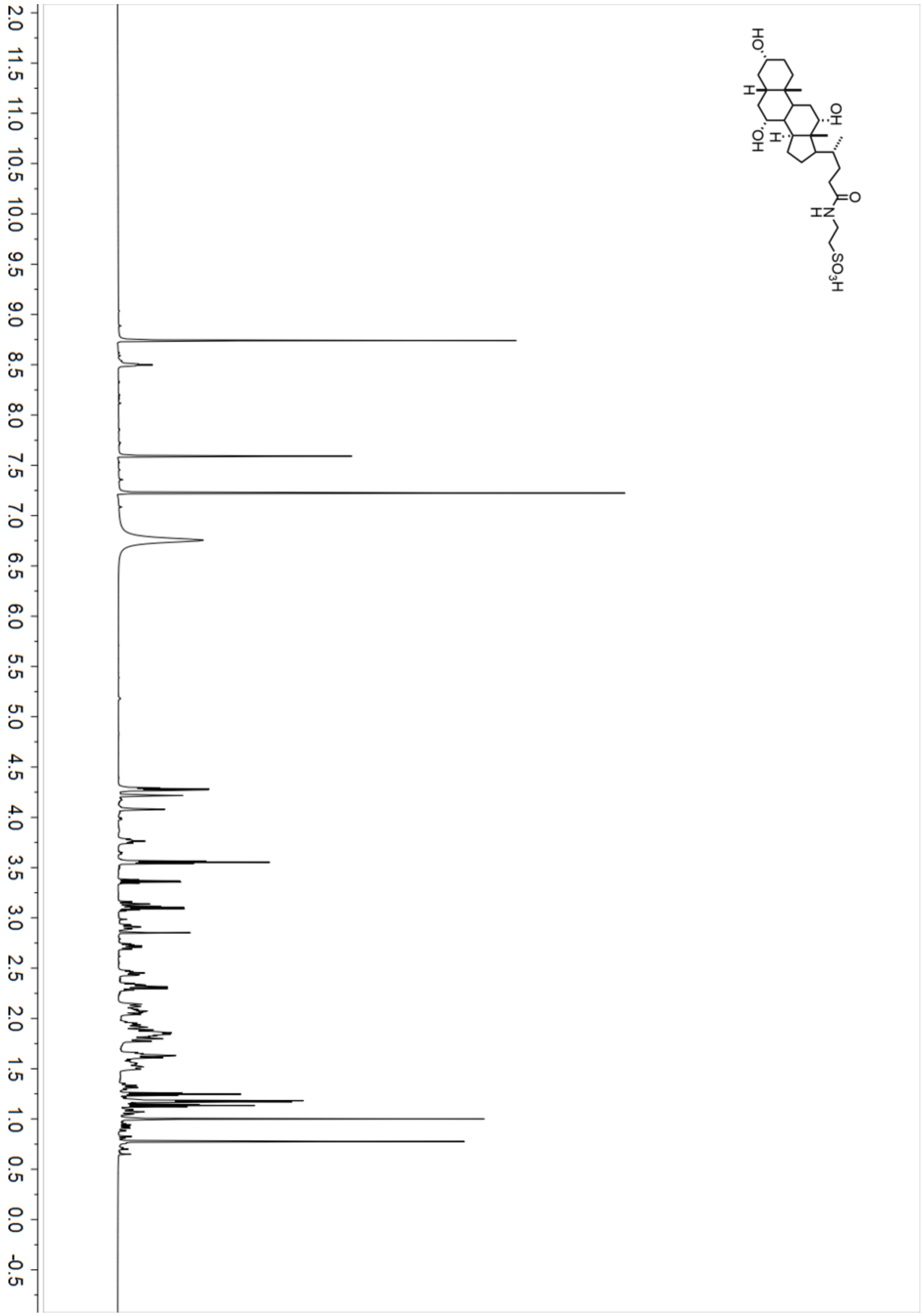

^{13}C NMR spectra of commercially purchased TCA (1)

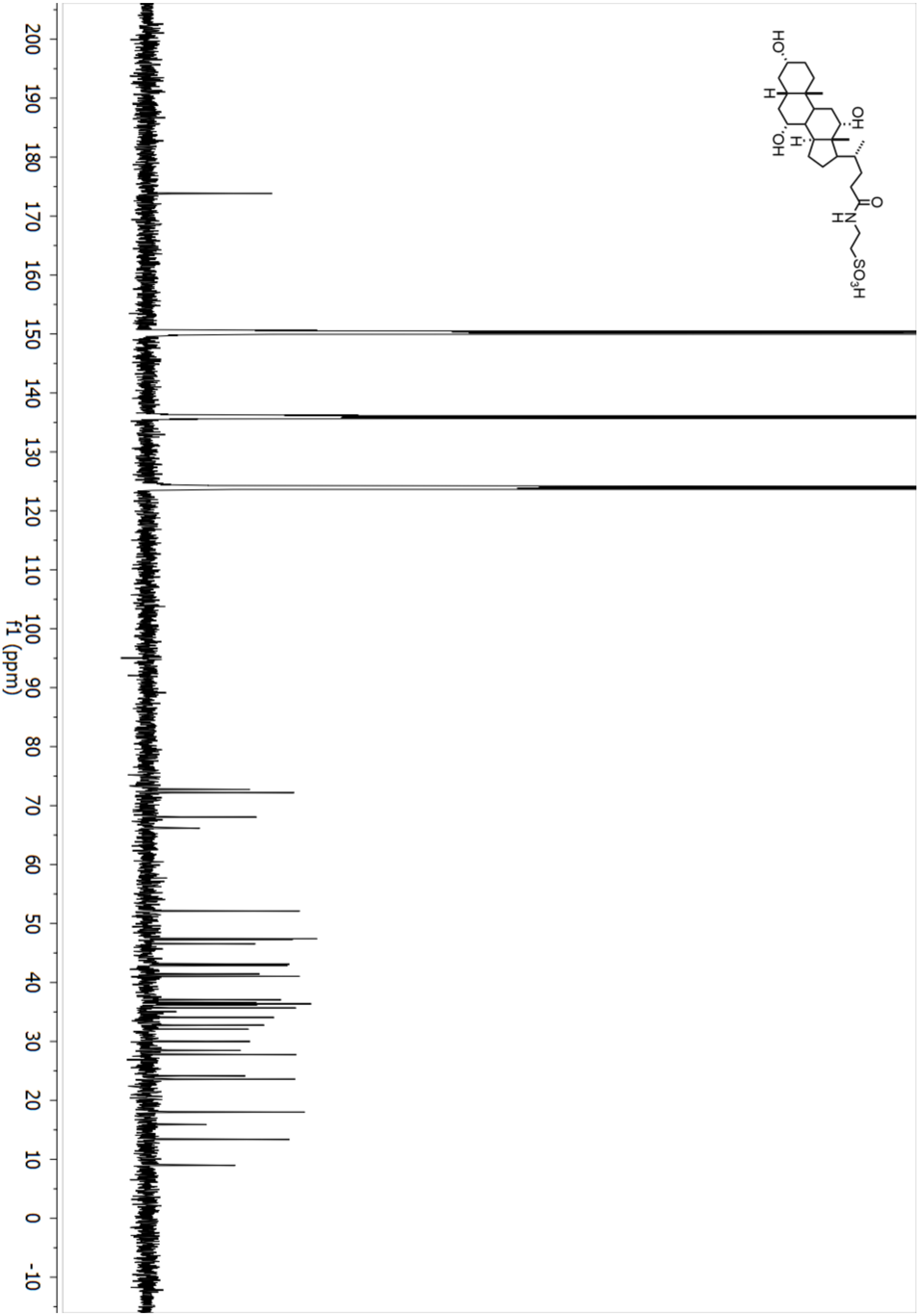

Supplement: S1 Fig — (PDF) [file pone.0149603.s001.pdf]
