# Supplementary material for: Biofilm Formation and Detachment in Gram-Negative Pathogens Is Modulated by Select Bile Acids
Source: PLoS One. 2016 Mar 18;11(3):e0149603. doi: 10.1371/journal.pone.0149603 (PMC4798295; doi:10.1371/journal.pone.0149603)

EIC of TCA (1) of commercially purchased sample of TCA (1) and that isolated from extract *R. erythropolis* FI1021DH2S2

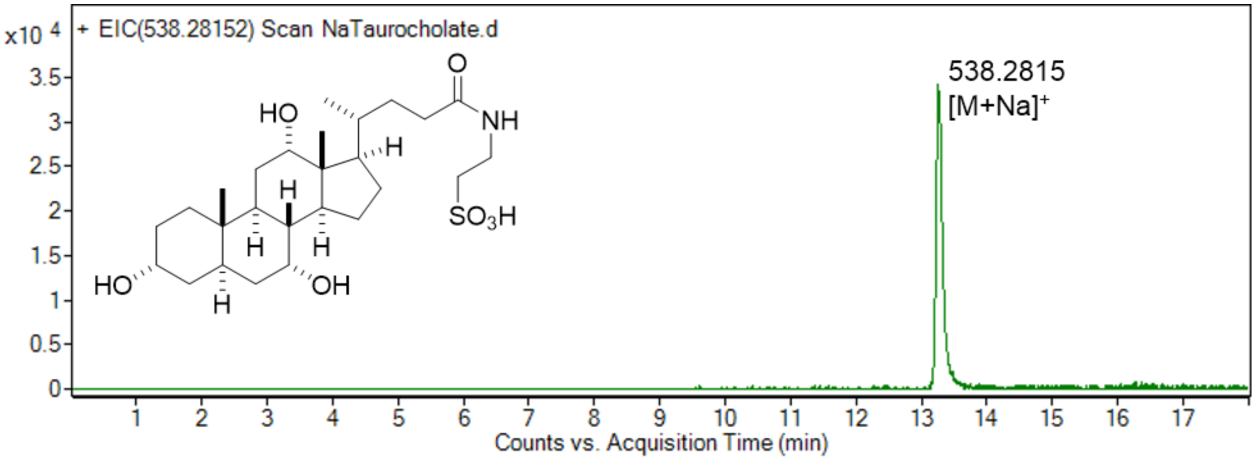

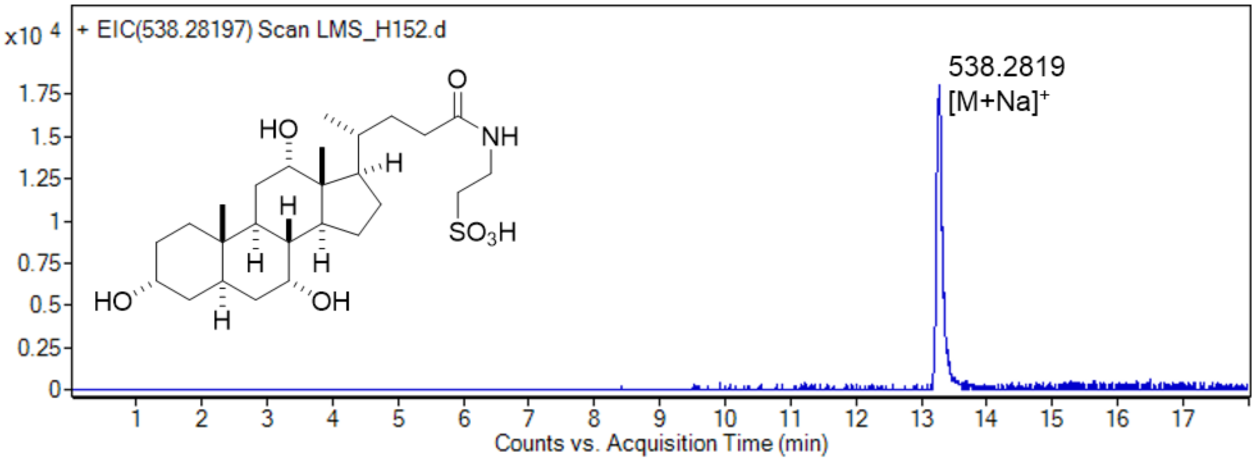

Supplement: S2 Fig — (PDF) [file pone.0149603.s002.pdf]
