# Supplementary material for: Biofilm Formation and Detachment in Gram-Negative Pathogens Is Modulated by Select Bile Acids
Source: PLoS One. 2016 Mar 18;11(3):e0149603. doi: 10.1371/journal.pone.0149603 (PMC4798295; doi:10.1371/journal.pone.0149603)

Figure S3. BIC_{50} curves for the active compounds against *V. cholerae*

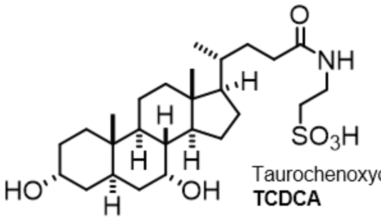

Taurochenoxydeoxycholic acid
TCDCA

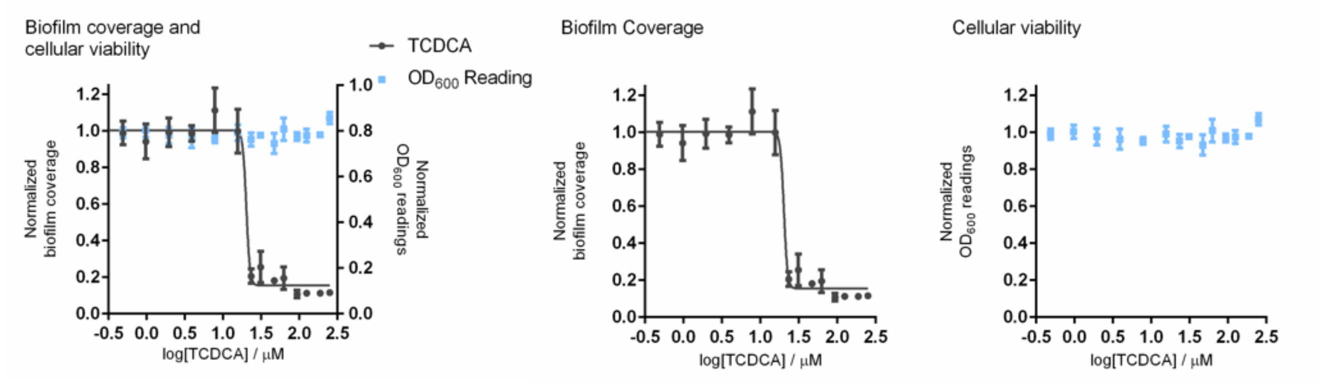

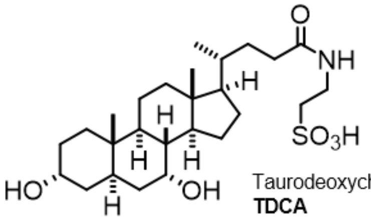

Taurodeoxycholic acid
TDCA

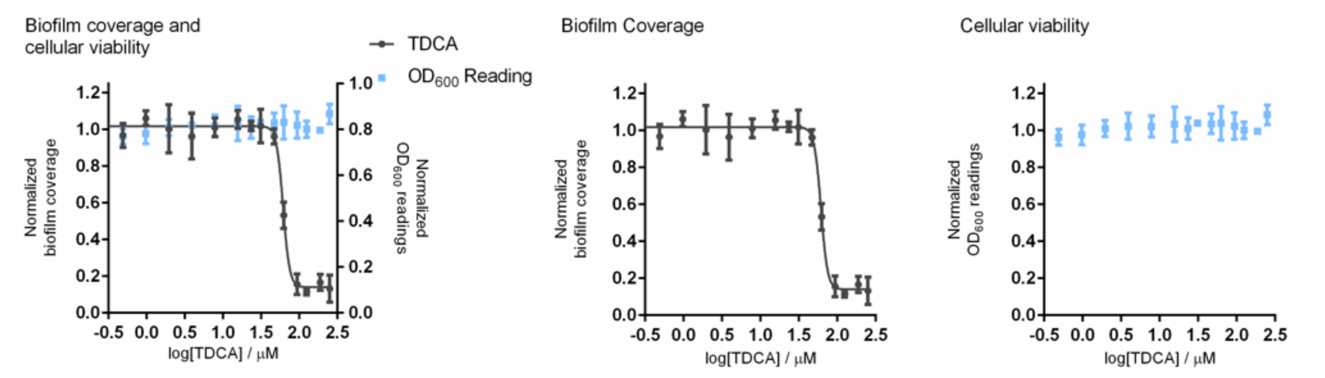

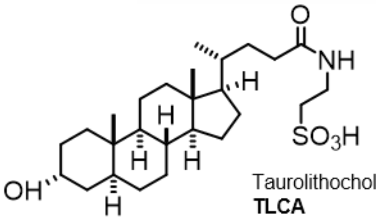

Taurolithocholic acid
TLCA

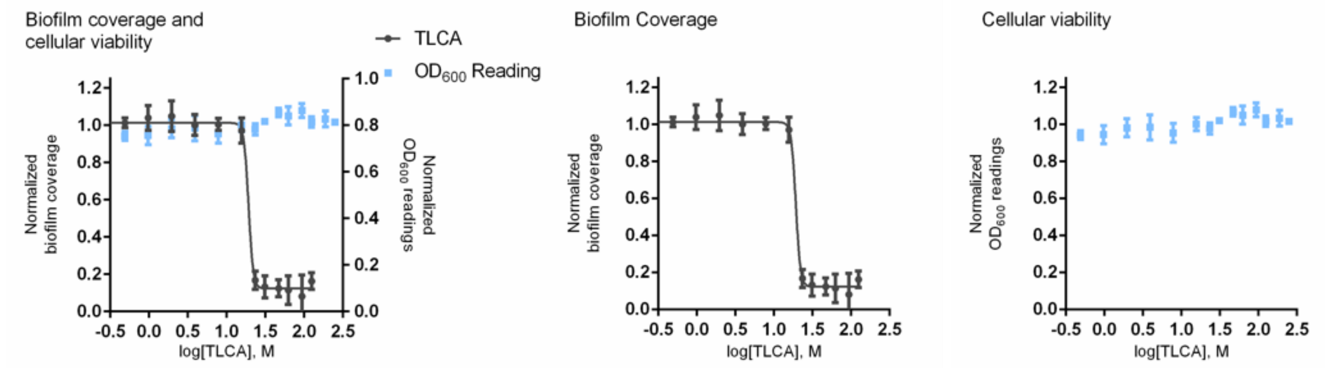

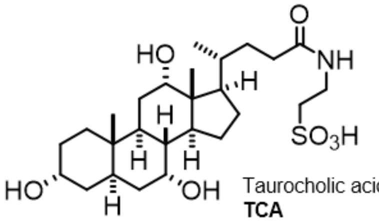

Taurocholic acid
TCA

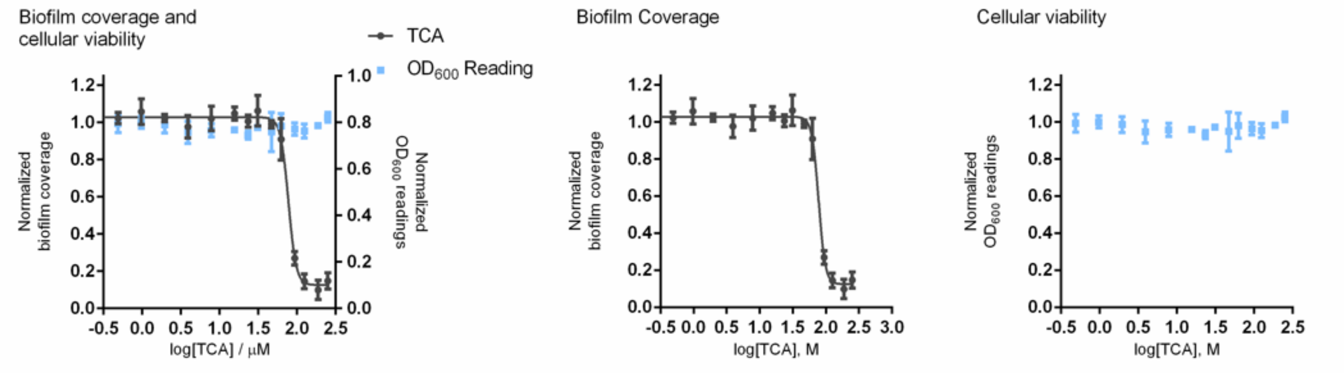

Supplement: S3 Fig — (PDF) [file pone.0149603.s003.pdf]
