# Supplementary material for: Biofilm Formation and Detachment in Gram-Negative Pathogens Is Modulated by Select Bile Acids
Source: PLoS One. 2016 Mar 18;11(3):e0149603. doi: 10.1371/journal.pone.0149603 (PMC4798295; doi:10.1371/journal.pone.0149603)

**Figure S4. Crystal Violet Assay of Biofilm Formation in *V. cholerae* Wild Type A1552 and *V. cholerae* sp. C6706 for Intact Bile and Individual Bile Acid Constituents.**

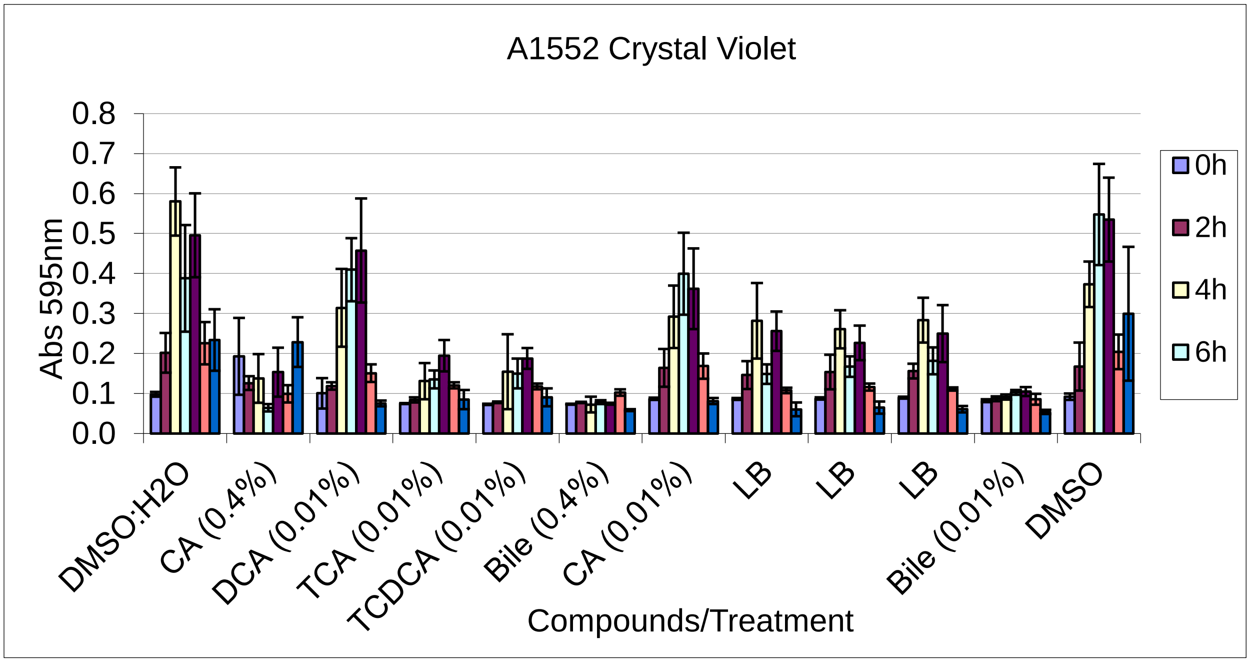


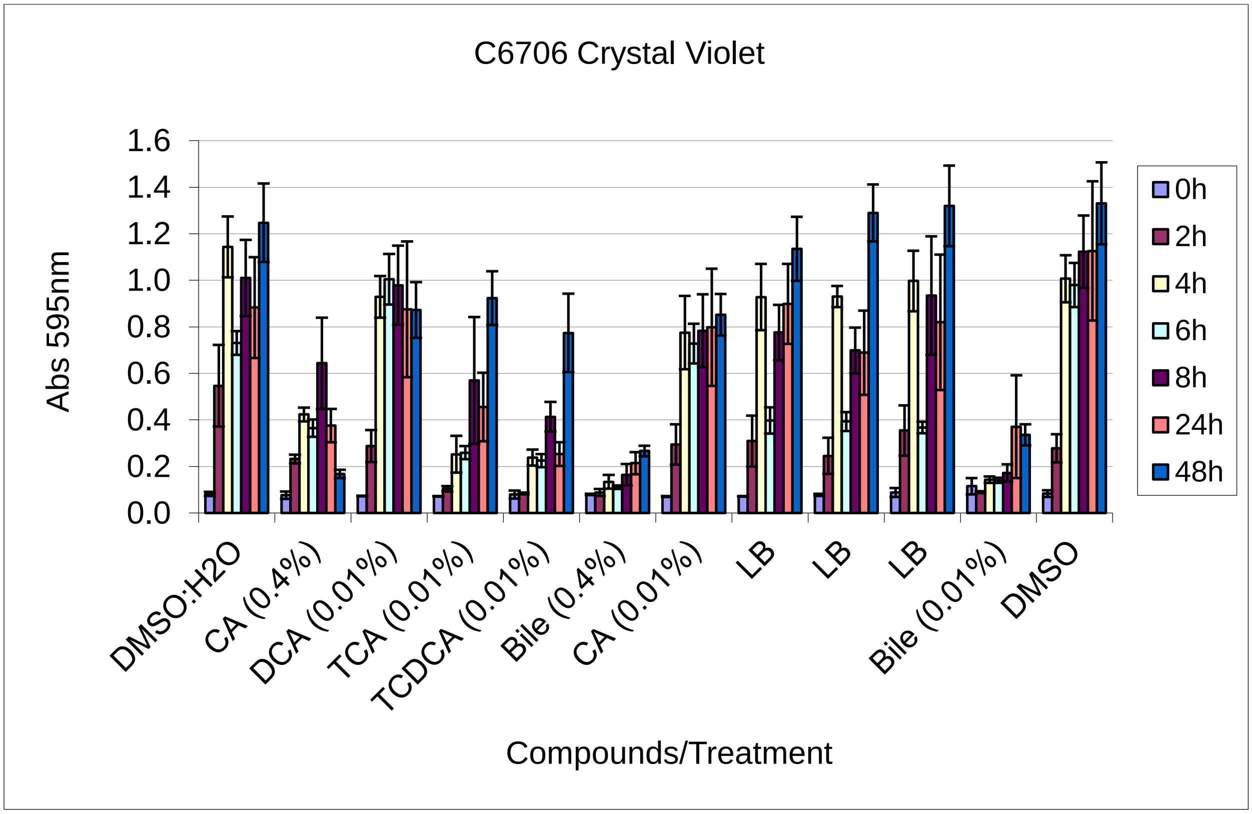

Supplement: S4 Fig — (PDF) [file pone.0149603.s004.pdf]
