# Supplementary figures and images for: Biofilm Formation and Detachment in Gram-Negative Pathogens Is Modulated by Select Bile Acids
Source: PLoS One. 2016 Mar 18;11(3):e0149603. doi: 10.1371/journal.pone.0149603 (PMC4798295; doi:10.1371/journal.pone.0149603)

**Figure S5** - BIC<sub>50</sub> curves for the active compounds against *P. aeruginosa*.

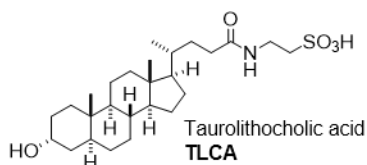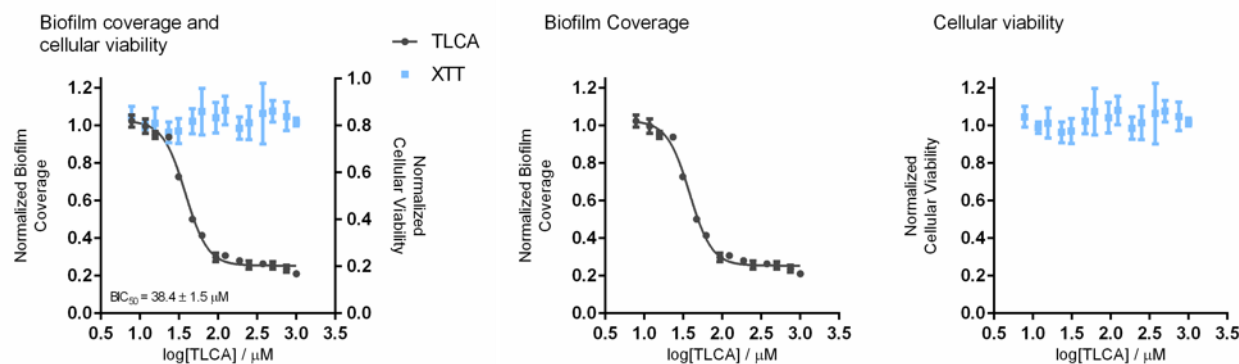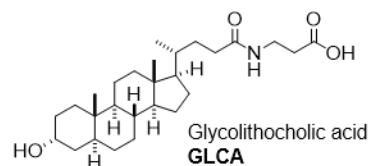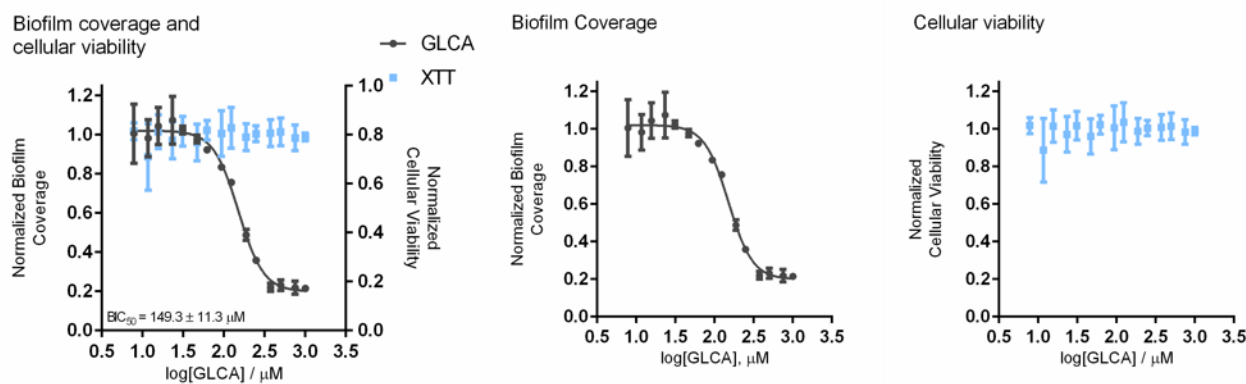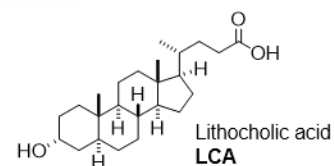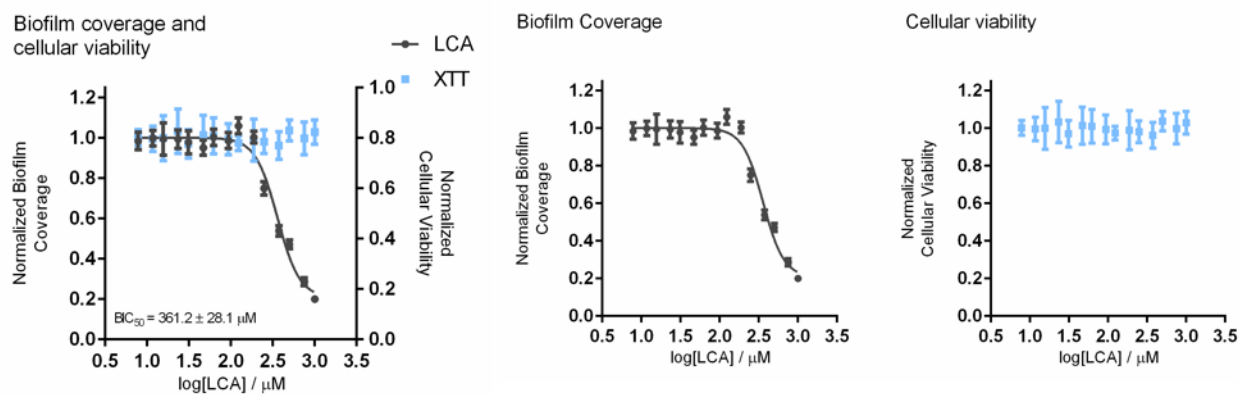

Supplement: S5 Fig — (PDF) [file pone.0149603.s005.pdf]
