# Supplementary material for: Biofilm Formation and Detachment in Gram-Negative Pathogens Is Modulated by Select Bile Acids
Source: PLoS One. 2016 Mar 18;11(3):e0149603. doi: 10.1371/journal.pone.0149603 (PMC4798295; doi:10.1371/journal.pone.0149603)
Supplement: S1 Table — (PDF) [file pone.0149603.s006.pdf]

Table S1. COMSTAT Analysis for bile acid components that reduce biofilm formation in *V. cholerae*.

Time (h)	Condition	Total biomass ($\mu m^3/\mu m^2$)	Thickness (μm)	
			Average	Maximum
24	DMSO	26.03 (3.2)	22.57 (1.9)	36.67 (0.7)
24	TCA (**1**)	7.66 (1.4)	10.27 (2.1)	35.49 (0.9)
24	TCDCA (**2**)	2.45 (0.8)	5.42 (1.8)	35.05 (0.7)
