# Supplementary material for: Biofilm Formation and Detachment in Gram-Negative Pathogens Is Modulated by Select Bile Acids
Source: PLoS One. 2016 Mar 18;11(3):e0149603. doi: 10.1371/journal.pone.0149603 (PMC4798295; doi:10.1371/journal.pone.0149603)
Supplement: S2 Table — (PDF) [file pone.0149603.s007.pdf]

**Table S2.** COMSTAT Analysis for *V. cholerae* preformed biofilm formation.

| Time (h) | Condition | Total biomass ($\mu m^3/\mu m^2$) | Thickness ($\mu$m) | |
|---|---|---|---|---|
| | | | Average | Maximum |
| 5 | No compound | 23.21 (3.0) | 28.54 (3.6) | 44.88 (4.0) |
| | DMSO | 20.89 (3.1) | 26.14 (5.0) | 41.65 (3.5) |
| 7 | No compound | 24.33 (1.2) | 21.96 (5.5) | 45.50 (5.9) |
| | DMSO | 23.96 (2.2) | 21.04 (3.2) | 43.88 (2.6) |
| | Bile | 19.13 (0.9) | 19.48 (1.1) | 33.73 (8.8) |
| | TCA (**1**) | 11.88 (0.4) | 12.91 (0.4) | 29.92 (3.8) |
| | TCDCA (**2**) | 8.70 (2.2) | 6.80 (3.7) | 29.62 (8.2) |
| 24 | No compound | 20.02 (3.0) | 21.96 (5.5) | 47.23 (3.1) |
| | DMSO | 19.96 (2.2) | 21.04 (3.2) | 42.88 (4.6) |
| | Bile | 16.00 (2.4) | 19.76 (3.0) | 40.48 (9.5) |
| | TCA (**1**) | 4.99 (0.3) | 7.63 (0.5) | 39.30 (7.6) |
| | TCDCA (**2**) | 0.89 (0.4) | 1.21(1.1) | 3.96 (5.7) |
