# Supplementary material for: Biofilm Formation and Detachment in Gram-Negative Pathogens Is Modulated by Select Bile Acids
Source: PLoS One. 2016 Mar 18;11(3):e0149603. doi: 10.1371/journal.pone.0149603 (PMC4798295; doi:10.1371/journal.pone.0149603)
Supplement: S3 Table — (PDF) [file pone.0149603.s008.pdf]

Table S3. COMSTAT Analysis for bile acid components that reduce biofilm formation in *P. aeruginosa*.

COMSTAT analysis of biofilm formation

Condition	Time (h)	Total biomass ($\mu m^3/\mu m^2$)	Thickness (μm)	
			Average	Maximum
DMSO	24	11.00 (1.8)	19.05 (2.0)	51.50 (5.0)
TLCA (**4**)	24	7.01 (1.4)	13.5 (2.0)	85.00 (17.8)

COMSTAT analysis of preformed biofilm formation

Condition	Time (h)	Total biomass ($\mu m^3/\mu m^2$)	Thickness (μm)	
			Average	Maximum
DMSO	24	23.00 (3.0)	33.65 (4.9)	71.85 (2.5)
TLCA (**4**)	24	20.10 (1.4)	18.15 (0.3)	30.80 (0.0)
