# Supplementary material for: Biofilm Formation and Detachment in Gram-Negative Pathogens Is Modulated by Select Bile Acids
Source: PLoS One. 2016 Mar 18;11(3):e0149603. doi: 10.1371/journal.pone.0149603 (PMC4798295; doi:10.1371/journal.pone.0149603)
Supplement: S4 Table — (PDF) [file pone.0149603.s009.pdf]

Table S4. Fatty acids and other major constituents of bile screened in our high content *V. cholerae* assay.

Compound	Reported MIC	Highest Conc Tested
palmitic acid	15 mM[a], 5mM[b]	1 mM
cis-palmitoleic acid	NA[c]	1 mM
stearic acid	14 mM[a]	250 µM
arachidonic acid	2.5 mM[a]	1 mM
linoleic acid	3.5 mM[a]	1 mM
oleic acid	2.7 mM[a]	1 mM
bilirubin	nt	250 µM
phosphatidylcholine	nt	250 µM

nt = not previously tested other studies.

a see reference 1.

b see reference 2.

c, see reference 3. Crystal structure obtained, no MIC reported.

References

1. Chatterjee, A.; Dutta, P. K.; Chowdhury, R. *Infect Immun* **2007,** *75* (4), 1946-1953.
2. Lowden, M. J.; Skorupski, K.; Pellegrini, M.; Chiorazzo, M. G.; Taylor, R. K.; Kull, F. J. *Proc Natl Acad Sci U.S.A.* **2010,** *107* (7), 2860-2865.
3. Ray, S.; Chatterjee, E.; Chatterjee, A.; Paul, K.; Chowdhury, R. *Infect Immun* **2011,** *79* (1), 258-266.
